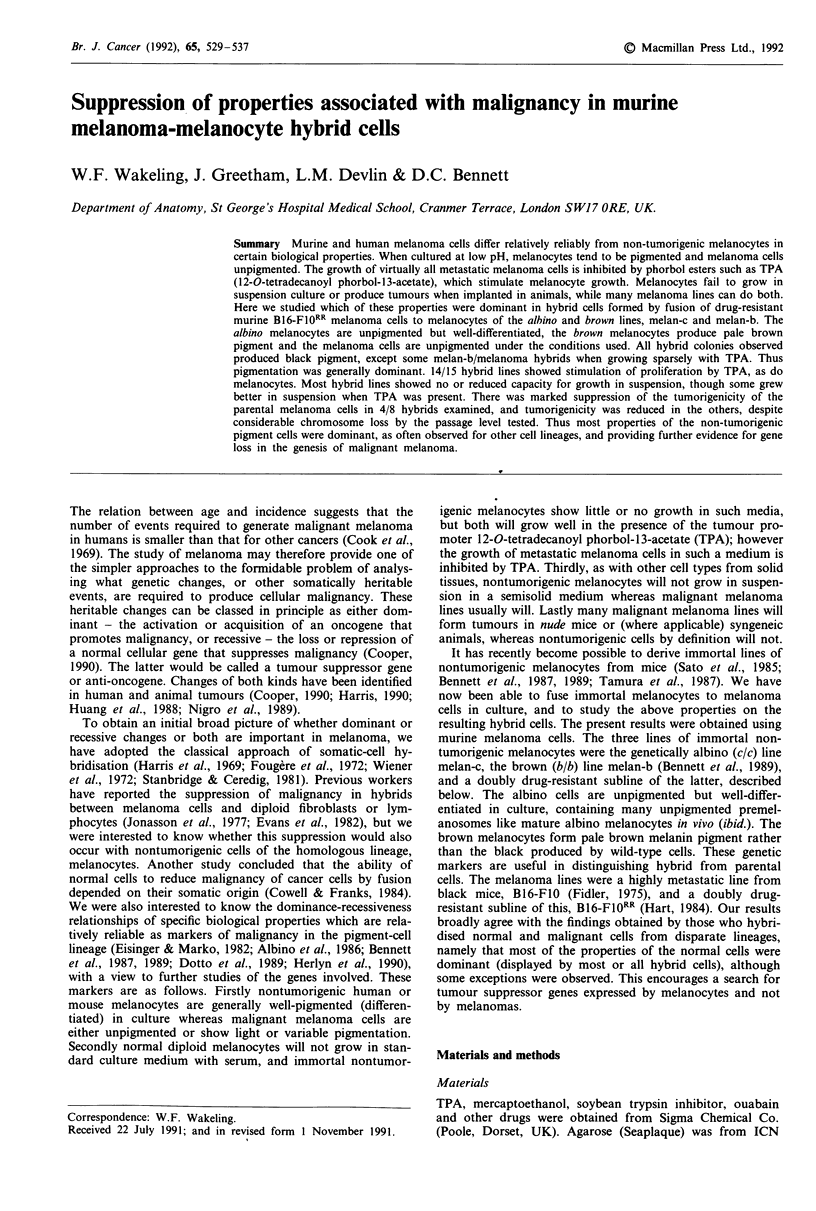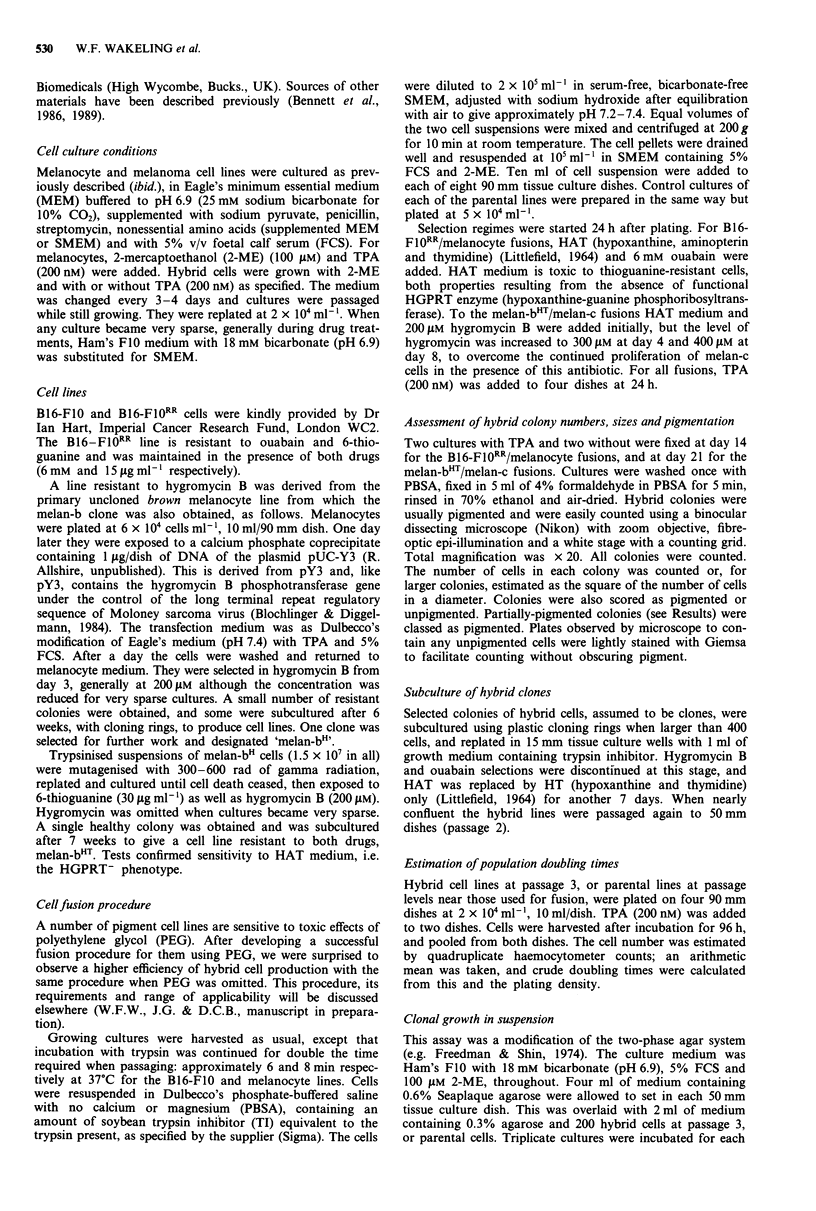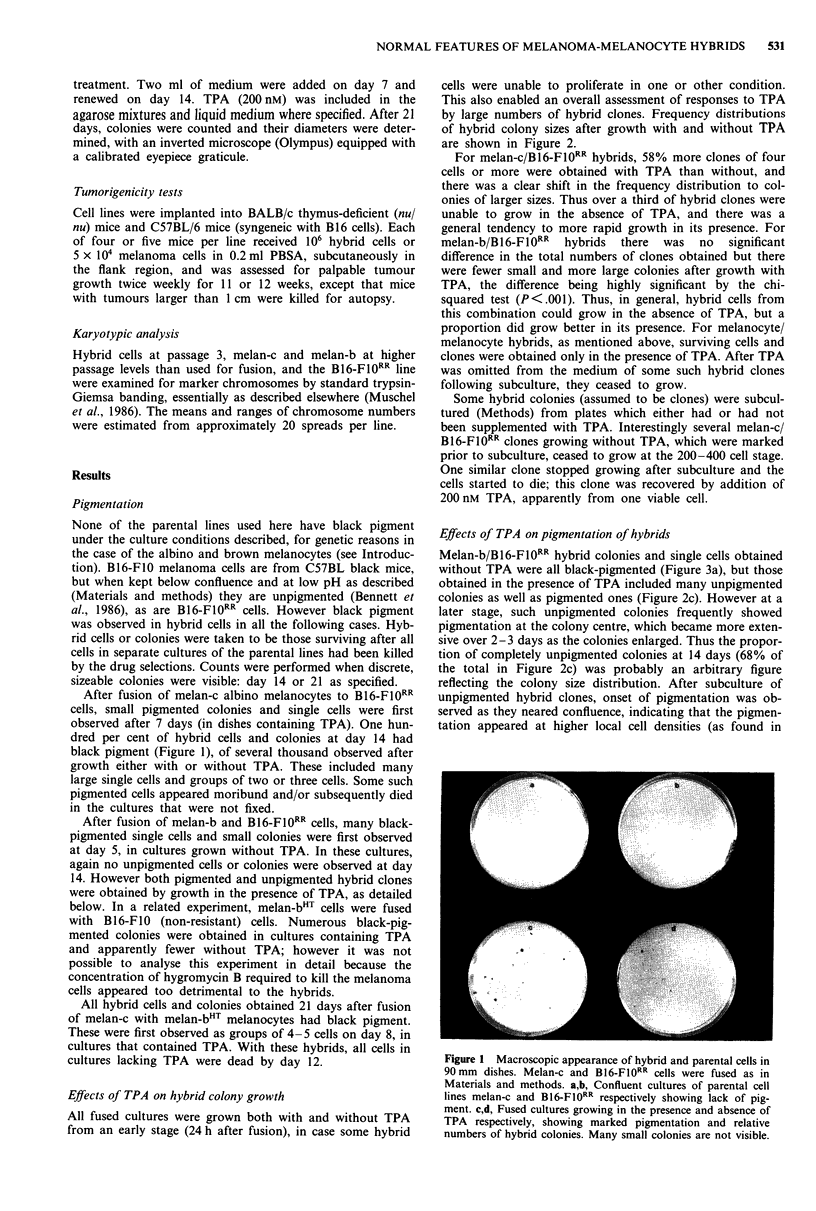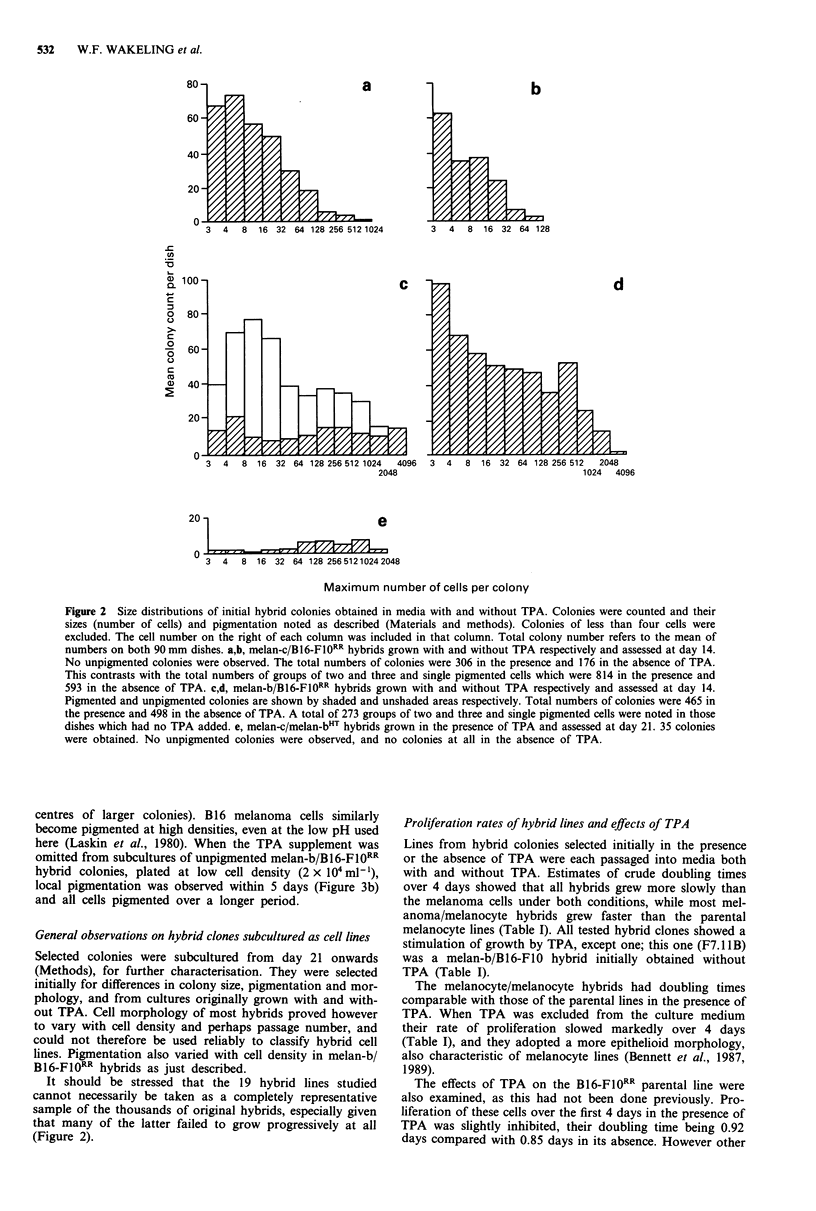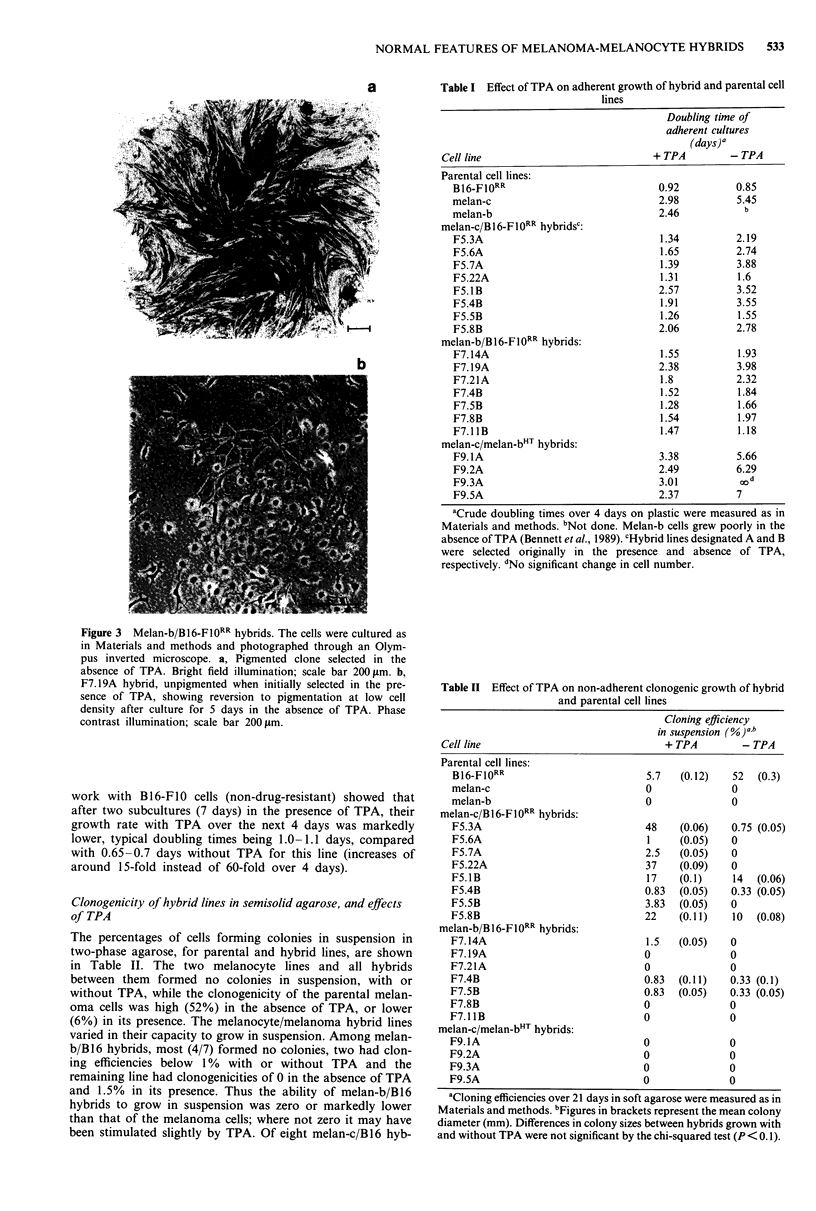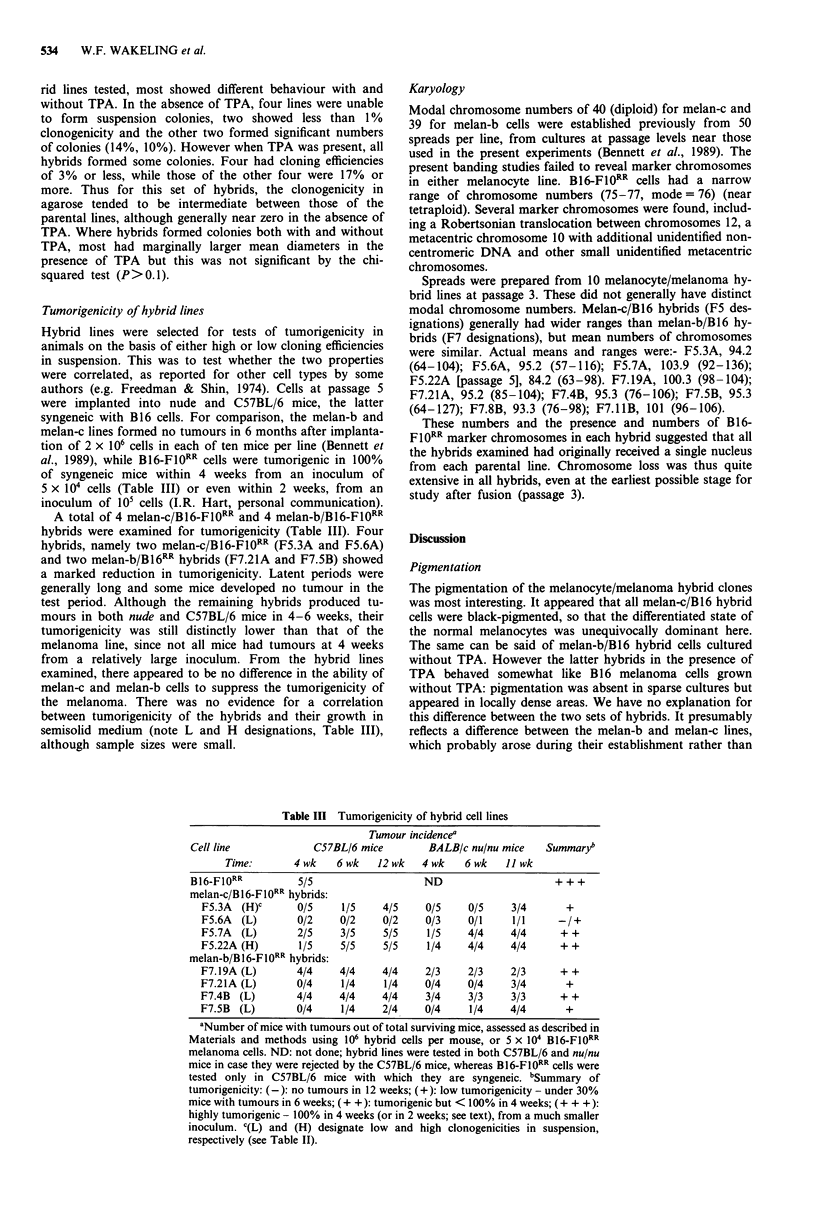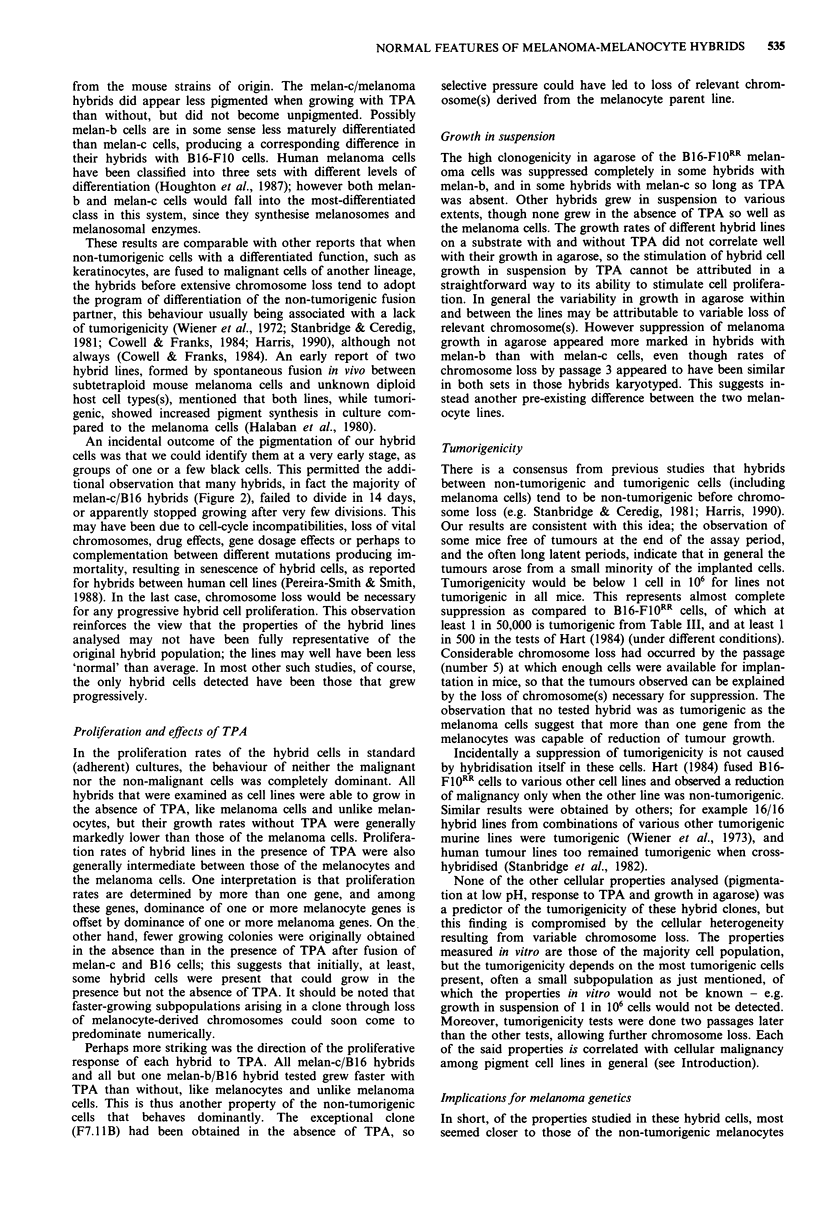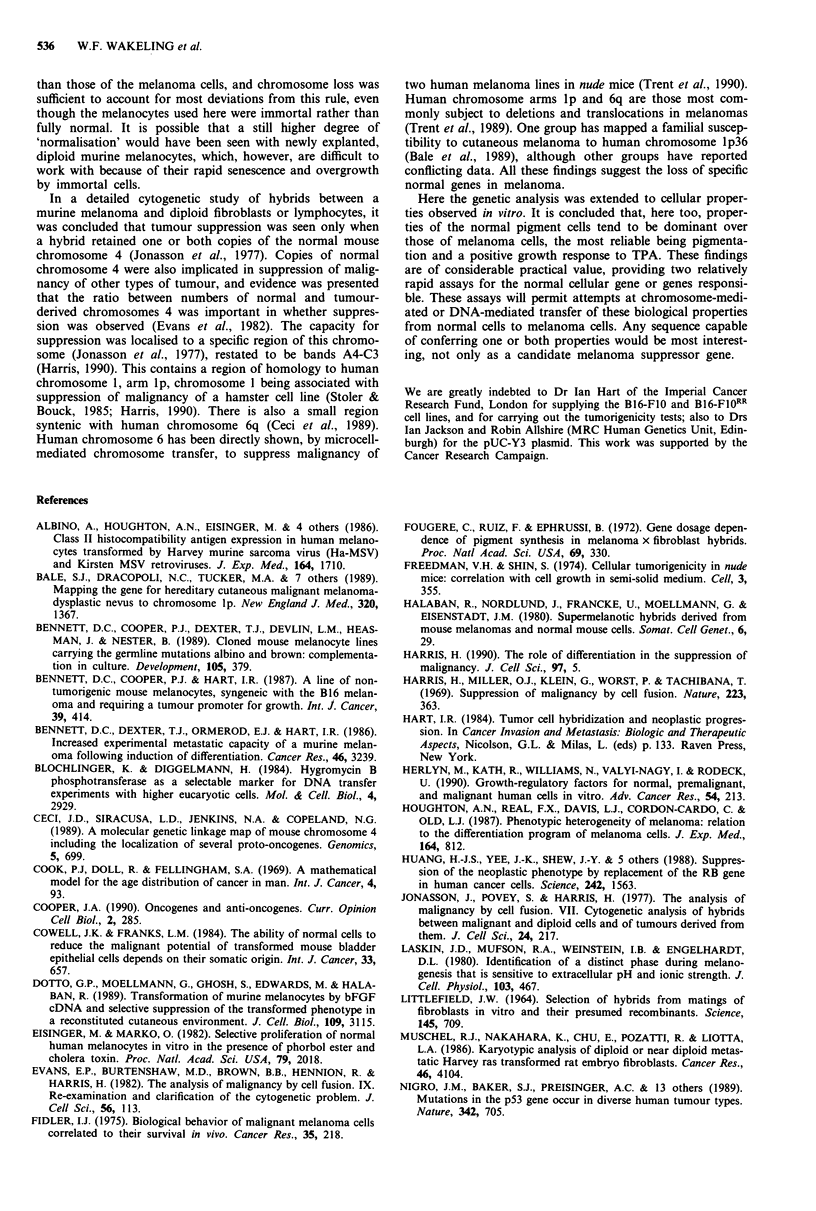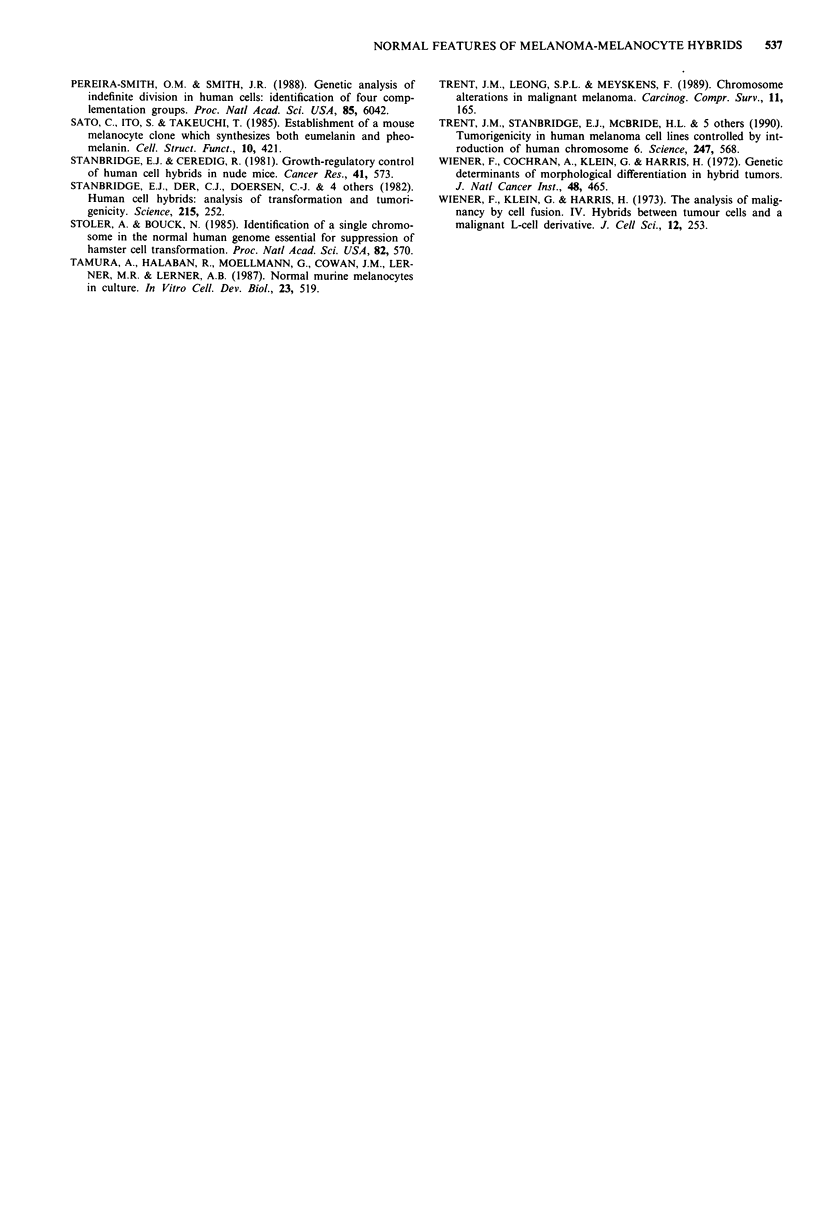# Suppression of properties associated with malignancy in murine melanoma-melanocyte hybrid cells.

**DOI:** 10.1038/bjc.1992.110

**Published:** 1992-04

**Authors:** W. F. Wakeling, J. Greetham, L. M. Devlin, D. C. Bennett

**Affiliations:** Department of Anatomy, St George's Hospital Medical School, London, UK.

## Abstract

**Images:**


Br. J. Cancer (1992), 65, 529-537 © Macmillan Press Ltd., 1992

### Suppression of properties associated with malignancy in murine melanoma-melanocyte hybrid cells

W.F. Wakeling, J. Greetham, L.M. Devlin & D.C. Bennett

*Department of Anatomy, St George's Hospital Medical School, Cranmer Terrace, London SW17 0RE, UK.*

**Summary** Murine and human melanoma cells differ relatively reliably from non-tumorigenic melanocytes in certain biological properties. When cultured at low pH, melanocytes tend to be pigmented and melanoma cells unpigmented. The growth of virtually all metastatic melanoma cells is inhibited by phorbol esters such as TPA (12-*O*-tetradecanoyl phorbol-13-acetate), which stimulate melanocyte growth. Melanocytes fail to grow in suspension culture or produce tumours when implanted in animals, while many melanoma lines can do both. Here we studied which of these properties were dominant in hybrid cells formed by fusion of drug-resistant murine B16-F10$^{RR}$ melanoma cells to melanocytes of the *albino* and *brown* lines, melan-c and melan-b. The *albino* melanocytes are unpigmented but well-differentiated, the *brown* melanocytes produce pale brown pigment and the melanoma cells are unpigmented under the conditions used. All hybrid colonies observed produced black pigment, except some melan-b/melanoma hybrids when growing sparsely with TPA. Thus pigmentation was generally dominant. 14/15 hybrid lines showed stimulation of proliferation by TPA, as do melanocytes. Most hybrid lines showed no or reduced capacity for growth in suspension, though some grew better in suspension when TPA was present. There was marked suppression of the tumorigenicity of the parental melanoma cells in 4/8 hybrids examined, and tumorigenicity was reduced in the others, despite considerable chromosome loss by the passage level tested. Thus most properties of the non-tumorigenic pigment cells were dominant, as often observed for other cell lineages, and providing further evidence for gene loss in the genesis of malignant melanoma.

The relation between age and incidence suggests that the number of events required to generate malignant melanoma in humans is smaller than that for other cancers (Cook *et al.*, 1969). The study of melanoma may therefore provide one of the simpler approaches to the formidable problem of analysing what genetic changes, or other somatically heritable events, are required to produce cellular malignancy. These heritable changes can be classed in principle as either dominant – the activation or acquisition of an oncogene that promotes malignancy, or recessive – the loss or repression of a normal cellular gene that suppresses malignancy (Cooper, 1990). The latter would be called a tumour suppressor gene or anti-oncogene. Changes of both kinds have been identified in human and animal tumours (Cooper, 1990; Harris, 1990; Huang *et al.*, 1988; Nigro *et al.*, 1989).

To obtain an initial broad picture of whether dominant or recessive changes or both are important in melanoma, we have adopted the classical approach of somatic-cell hybridisation (Harris *et al.*, 1969; Fougère *et al.*, 1972; Wiener *et al.*, 1972; Stanbridge & Ceredig, 1981). Previous workers have reported the suppression of malignancy in hybrids between melanoma cells and diploid fibroblasts or lymphocytes (Jonasson *et al.*, 1977; Evans *et al.*, 1982), but we were interested to know whether this suppression would also occur with nontumorigenic cells of the homologous lineage, melanocytes. Another study concluded that the ability of normal cells to reduce malignancy of cancer cells by fusion depended on their somatic origin (Cowell & Franks, 1984). We were also interested to know the dominance-recessiveness relationships of specific biological properties which are relatively reliable as markers of malignancy in the pigment-cell lineage (Eisinger & Marko, 1982; Albino *et al.*, 1986; Bennett *et al.*, 1987, 1989; Dotto *et al.*, 1989; Herlyn *et al.*, 1990), with a view to further studies of the genes involved. These markers are as follows. Firstly nontumorigenic human or mouse melanocytes are generally well-pigmented (differentiated) in culture whereas malignant melanoma cells are either unpigmented or show light or variable pigmentation. Secondly normal diploid melanocytes will not grow in standard culture medium with serum, and immortal nontumorigenic melanocytes show little or no growth in such media, but both will grow well in the presence of the tumour promoter 12-*O*-tetradecanoyl phorbol-13-acetate (TPA); however the growth of metastatic melanoma cells in such a medium is inhibited by TPA. Thirdly, as with other cell types from solid tissues, nontumorigenic melanocytes will not grow in suspension in a semisolid medium whereas malignant melanoma lines usually will. Lastly many malignant melanoma lines will form tumours in *nude* mice or (where applicable) syngeneic animals, whereas nontumorigenic cells by definition will not.

It has recently become possible to derive immortal lines of nontumorigenic melanocytes from mice (Sato *et al.*, 1985; Bennett *et al.*, 1987, 1989; Tamura *et al.*, 1987). We have now been able to fuse immortal melanocytes to melanoma cells in culture, and to study the above properties on the resulting hybrid cells. The present results were obtained using murine melanoma cells. The three lines of immortal nontumorigenic melanocytes were the genetically albino (*c*/*c*) line melan-c, the brown (*b*/*b*) line melan-b (Bennett *et al.*, 1989), and a doubly drug-resistant subline of the latter, described below. The albino cells are unpigmented but well-differentiated in culture, containing many unpigmented premelanosomes like mature albino melanocytes *in vivo* (*ibid.*). The brown melanocytes form pale brown melanin pigment rather than the black produced by wild-type cells. These genetic markers are useful in distinguishing hybrid from parental cells. The melanoma lines were a highly metastatic line from black mice, B16-F10 (Fidler, 1975), and a doubly drug-resistant subline of this, B16-F10$^{RR}$ (Hart, 1984). Our results broadly agree with the findings obtained by those who hybridised normal and malignant cells from disparate lineages, namely that most of the properties of the normal cells were dominant (displayed by most or all hybrid cells), although some exceptions were observed. This encourages a search for tumour suppressor genes expressed by melanocytes and not by melanomas.

### Materials and methods

### *Materials*

TPA, mercaptoethanol, soybean trypsin inhibitor, ouabain and other drugs were obtained from Sigma Chemical Co. (Poole, Dorset, UK). Agarose (Seaplaque) was from ICN

Correspondence: W.F. Wakeling.
Received 22 July 1991; and in revised form 1 November 1991.

Biomedicals (High Wycombe, Bucks., UK). Sources of other materials have been described previously (Bennett et al., 1986, 1989).

### Cell culture conditions

Melanocyte and melanoma cell lines were cultured as previously described (ibid.), in Eagle's minimum essential medium (MEM) buffered to pH 6.9 (25 mM sodium bicarbonate for 10% $CO_2$), supplemented with sodium pyruvate, penicillin, streptomycin, nonessential amino acids (supplemented MEM or SMEM) and with 5% v/v foetal calf serum (FCS). For melanocytes, 2-mercaptoethanol (2-ME) (100 $\mu$M) and TPA (200 nM) were added. Hybrid cells were grown with 2-ME and with or without TPA (200 nM) as specified. The medium was changed every 3–4 days and cultures were passaged while still growing. They were replated at $2 \times 10^4$ $ml^{-1}$. When any culture became very sparse, generally during drug treatments, Ham's F10 medium with 18 mM bicarbonate (pH 6.9) was substituted for SMEM.

### Cell lines

B16-F10 and B16-F10$^{RR}$ cells were kindly provided by Dr Ian Hart, Imperial Cancer Research Fund, London WC2. The B16–F10$^{RR}$ line is resistant to ouabain and 6-thioguanine and was maintained in the presence of both drugs (6 mM and 15 $\mu g\ ml^{-1}$ respectively).

A line resistant to hygromycin B was derived from the primary uncloned *brown* melanocyte line from which the melan-b clone was also obtained, as follows. Melanocytes were plated at $6 \times 10^4$ cells $ml^{-1}$, 10 ml/90 mm dish. One day later they were exposed to a calcium phosphate coprecipitate containing 1 $\mu$g/dish of DNA of the plasmid pUC-Y3 (R. Allshire, unpublished). This is derived from pY3 and, like pY3, contains the hygromycin B phosphotransferase gene under the control of the long terminal repeat regulatory sequence of Moloney sarcoma virus (Blochlinger & Diggelmann, 1984). The transfection medium was as Dulbecco's modification of Eagle's medium (pH 7.4) with TPA and 5% FCS. After a day the cells were washed and returned to melanocyte medium. They were selected in hygromycin B from day 3, generally at 200 $\mu$M although the concentration was reduced for very sparse cultures. A small number of resistant colonies were obtained, and some were subcultured after 6 weeks, with cloning rings, to produce cell lines. One clone was selected for further work and designated 'melan-b$^{H}$'.

Trypsinised suspensions of melan-b$^{H}$ cells ($1.5 \times 10^7$ in all) were mutagenised with 300–600 rad of gamma radiation, replated and cultured until cell death ceased, then exposed to 6-thioguanine (30 $\mu g\ ml^{-1}$) as well as hygromycin B (200 $\mu$M). Hygromycin was omitted when cultures became very sparse. A single healthy colony was obtained and was subcultured after 7 weeks to give a cell line resistant to both drugs, melan-b$^{HT}$. Tests confirmed sensitivity to HAT medium, i.e. the HGPRT$^{-}$ phenotype.

### Cell fusion procedure

A number of pigment cell lines are sensitive to toxic effects of polyethylene glycol (PEG). After developing a successful fusion procedure for them using PEG, we were surprised to observe a higher efficiency of hybrid cell production with the same procedure when PEG was omitted. This procedure, its requirements and range of applicability will be discussed elsewhere (W.F.W., J.G. & D.C.B., manuscript in preparation).

Growing cultures were harvested as usual, except that incubation with trypsin was continued for double the time required when passaging: approximately 6 and 8 min respectively at 37°C for the B16-F10 and melanocyte lines. Cells were resuspended in Dulbecco's phosphate-buffered saline with no calcium or magnesium (PBSA), containing an amount of soybean trypsin inhibitor (TI) equivalent to the trypsin present, as specified by the supplier (Sigma). The cells were diluted to $2 \times 10^5$ $ml^{-1}$ in serum-free, bicarbonate-free SMEM, adjusted with sodium hydroxide after equilibration with air to give approximately pH 7.2–7.4. Equal volumes of the two cell suspensions were mixed and centrifuged at 200 *g* for 10 min at room temperature. The cell pellets were drained well and resuspended at $10^5$ $ml^{-1}$ in SMEM containing 5% FCS and 2-ME. Ten ml of cell suspension were added to each of eight 90 mm tissue culture dishes. Control cultures of each of the parental lines were prepared in the same way but plated at $5 \times 10^4$ $ml^{-1}$.

Selection regimes were started 24 h after plating. For B16-F10$^{RR}$/melanocyte fusions, HAT (hypoxanthine, aminopterin and thymidine) (Littlefield, 1964) and 6 mM ouabain were added. HAT medium is toxic to thioguanine-resistant cells, both properties resulting from the absence of functional HGPRT enzyme (hypoxanthine-guanine phosphoribosyltransferase). To the melan-b$^{HT}$/melan-c fusions HAT medium and 200 $\mu$M hygromycin B were added initially, but the level of hygromycin was increased to 300 $\mu$M at day 4 and 400 $\mu$M at day 8, to overcome the continued proliferation of melan-c cells in the presence of this antibiotic. For all fusions, TPA (200 nM) was added to four dishes at 24 h.

### Assessment of hybrid colony numbers, sizes and pigmentation

Two cultures with TPA and two without were fixed at day 14 for the B16-F10$^{RR}$/melanocyte fusions, and at day 21 for the melan-b$^{HT}$/melan-c fusions. Cultures were washed once with PBSA, fixed in 5 ml of 4% formaldehyde in PBSA for 5 min, rinsed in 70% ethanol and air-dried. Hybrid colonies were usually pigmented and were easily counted using a binocular dissecting microscope (Nikon) with zoom objective, fibreoptic epi-illumination and a white stage with a counting grid. Total magnification was × 20. All colonies were counted. The number of cells in each colony was counted or, for larger colonies, estimated as the square of the number of cells in a diameter. Colonies were also scored as pigmented or unpigmented. Partially-pigmented colonies (see Results) were classed as pigmented. Plates observed by microscope to contain any unpigmented cells were lightly stained with Giemsa to facilitate counting without obscuring pigment.

### Subculture of hybrid clones

Selected colonies of hybrid cells, assumed to be clones, were subcultured using plastic cloning rings when larger than 400 cells, and replated in 15 mm tissue culture wells with 1 ml of growth medium containing trypsin inhibitor. Hygromycin B and ouabain selections were discontinued at this stage, and HAT was replaced by HT (hypoxanthine and thymidine) only (Littlefield, 1964) for another 7 days. When nearly confluent the hybrid lines were passaged again to 50 mm dishes (passage 2).

### Estimation of population doubling times

Hybrid cell lines at passage 3, or parental lines at passage levels near those used for fusion, were plated on four 90 mm dishes at $2 \times 10^4$ $ml^{-1}$, 10 ml/dish. TPA (200 nM) was added to two dishes. Cells were harvested after incubation for 96 h, and pooled from both dishes. The cell number was estimated by quadruplicate haemocytometer counts; an arithmetic mean was taken, and crude doubling times were calculated from this and the plating density.

### Clonal growth in suspension

This assay was a modification of the two-phase agar system (e.g. Freedman & Shin, 1974). The culture medium was Ham's F10 with 18 mM bicarbonate (pH 6.9), 5% FCS and 100 $\mu$M 2-ME, throughout. Four ml of medium containing 0.6% Seaplaque agarose were allowed to set in each 50 mm tissue culture dish. This was overlaid with 2 ml of medium containing 0.3% agarose and 200 hybrid cells at passage 3, or parental cells. Triplicate cultures were incubated for each treatment. Two ml of medium were added on day 7 and renewed on day 14. TPA (200 nM) was included in the agarose mixtures and liquid medium where specified. After 21 days, colonies were counted and their diameters were determined, with an inverted microscope (Olympus) equipped with a calibrated eyepiece graticule.

*Tumorigenicity tests*

Cell lines were implanted into BALB/c thymus-deficient (*nu/nu*) mice and C57BL/6 mice (syngeneic with B16 cells). Each of four or five mice per line received $10^6$ hybrid cells or $5 \times 10^4$ melanoma cells in 0.2 ml PBSA, subcutaneously in the flank region, and was assessed for palpable tumour growth twice weekly for 11 or 12 weeks, except that mice with tumours larger than 1 cm were killed for autopsy.

*Karyotypic analysis*

Hybrid cells at passage 3, melan-c and melan-b at higher passage levels than used for fusion, and the B16-F10$^{RR}$ line were examined for marker chromosomes by standard trypsin-Giemsa banding, essentially as described elsewhere (Muschel *et al.*, 1986). The means and ranges of chromosome numbers were estimated from approximately 20 spreads per line.

### Results

*Pigmentation*

None of the parental lines used here have black pigment under the culture conditions described, for genetic reasons in the case of the albino and brown melanocytes (see Introduction). B16-F10 melanoma cells are from C57BL black mice, but when kept below confluence and at low pH as described (Materials and methods) they are unpigmented (Bennett *et al.*, 1986), as are B16-F10$^{RR}$ cells. However black pigment was observed in hybrid cells in all the following cases. Hybrid cells or colonies were taken to be those surviving after all cells in separate cultures of the parental lines had been killed by the drug selections. Counts were performed when discrete, sizeable colonies were visible: day 14 or 21 as specified.

After fusion of melan-c albino melanocytes to B16-F10$^{RR}$ cells, small pigmented colonies and single cells were first observed after 7 days (in dishes containing TPA). One hundred per cent of hybrid cells and colonies at day 14 had black pigment (Figure 1), of several thousand observed after growth either with or without TPA. These included many large single cells and groups of two or three cells. Some such pigmented cells appeared moribund and/or subsequently died in the cultures that were not fixed.

After fusion of melan-b and B16-F10$^{RR}$ cells, many black-pigmented single cells and small colonies were first observed at day 5, in cultures grown without TPA. In these cultures, again no unpigmented cells or colonies were observed at day 14. However both pigmented and unpigmented hybrid clones were obtained by growth in the presence of TPA, as detailed below. In a related experiment, melan-b$^{HT}$ cells were fused with B16-F10 (non-resistant) cells. Numerous black-pigmented colonies were obtained in cultures containing TPA and apparently fewer without TPA; however it was not possible to analyse this experiment in detail because the concentration of hygromycin B required to kill the melanoma cells appeared too detrimental to the hybrids.

All hybrid cells and colonies obtained 21 days after fusion of melan-c with melan-b$^{HT}$ melanocytes had black pigment. These were first observed as groups of 4–5 cells on day 8, in cultures that contained TPA. With these hybrids, all cells in cultures lacking TPA were dead by day 12.

*Effects of TPA on hybrid colony growth*

All fused cultures were grown both with and without TPA from an early stage (24 h after fusion), in case some hybrid cells were unable to proliferate in one or other condition. This also enabled an overall assessment of responses to TPA by large numbers of hybrid clones. Frequency distributions of hybrid colony sizes after growth with and without TPA are shown in Figure 2.

For melan-c/B16-F10$^{RR}$ hybrids, 58% more clones of four cells or more were obtained with TPA than without, and there was a clear shift in the frequency distribution to colonies of larger sizes. Thus over a third of hybrid clones were unable to grow in the absence of TPA, and there was a general tendency to more rapid growth in its presence. For melan-b/B16-F10$^{RR}$ hybrids there was no significant difference in the total numbers of clones obtained but there were fewer small and more large colonies after growth with TPA, the difference being highly significant by the chi-squared test ($P < .001$). Thus, in general, hybrid cells from this combination could grow in the absence of TPA, but a proportion did grow better in its presence. For melanocyte/melanocyte hybrids, as mentioned above, surviving cells and clones were obtained only in the presence of TPA. After TPA was omitted from the medium of some such hybrid clones following subculture, they ceased to grow.

Some hybrid colonies (assumed to be clones) were subcultured (Methods) from plates which either had or had not been supplemented with TPA. Interestingly several melan-c/B16-F10$^{RR}$ clones growing without TPA, which were marked prior to subculture, ceased to grow at the 200–400 cell stage. One similar clone stopped growing after subculture and the cells started to die; this clone was recovered by addition of 200 nM TPA, apparently from one viable cell.

*Effects of TPA on pigmentation of hybrids*

Melan-b/B16-F10$^{RR}$ hybrid colonies and single cells obtained without TPA were all black-pigmented (Figure 3a), but those obtained in the presence of TPA included many unpigmented colonies as well as pigmented ones (Figure 2c). However at a later stage, such unpigmented colonies frequently showed pigmentation at the colony centre, which became more extensive over 2–3 days as the colonies enlarged. Thus the proportion of completely unpigmented colonies at 14 days (68% of the total in Figure 2c) was probably an arbitrary figure reflecting the colony size distribution. After subculture of unpigmented hybrid clones, onset of pigmentation was observed as they neared confluence, indicating that the pigmentation appeared at higher local cell densities (as found in

**Figure 1** Macroscopic appearance of hybrid and parental cells in 90 mm dishes. Melan-c and B16-F10$^{RR}$ cells were fused as in Materials and methods. **a**,**b**, Confluent cultures of parental cell lines melan-c and B16-F10$^{RR}$ respectively showing lack of pigment. **c**,**d**, Fused cultures growing in the presence and absence of TPA respectively, showing marked pigmentation and relative numbers of hybrid colonies. Many small colonies are not visible.

**Figure 2** Size distributions of initial hybrid colonies obtained in media with and without TPA. Colonies were counted and their sizes (number of cells) and pigmentation noted as described (Materials and methods). Colonies of less than four cells were excluded. The cell number on the right of each column was included in that column. Total colony number refers to the mean of numbers on both 90 mm dishes. **a**,**b**, melan-c/B16-F10$^{RR}$ hybrids grown with and without TPA respectively and assessed at day 14. No unpigmented colonies were observed. The total numbers of colonies were 306 in the presence and 176 in the absence of TPA. This contrasts with the total numbers of groups of two and three and single pigmented cells which were 814 in the presence and 593 in the absence of TPA. **c**,**d**, melan-b/B16-F10$^{RR}$ hybrids grown with and without TPA respectively and assessed at day 14. Pigmented and unpigmented colonies are shown by shaded and unshaded areas respectively. Total numbers of colonies were 465 in the presence and 498 in the absence of TPA. A total of 273 groups of two and three and single pigmented cells were noted in those dishes which had no TPA added. **e**, melan-c/melan-b$^{HT}$ hybrids grown in the presence of TPA and assessed at day 21. 35 colonies were obtained. No unpigmented colonies were observed, and no colonies at all in the absence of TPA.

centres of larger colonies). B16 melanoma cells similarly become pigmented at high densities, even at the low pH used here (Laskin *et al.*, 1980). When the TPA supplement was omitted from subcultures of unpigmented melan-b/B16-F10$^{RR}$ hybrid colonies, plated at low cell density ($2 \times 10^4 ml^{-1}$), local pigmentation was observed within 5 days (Figure 3b) and all cells pigmented over a longer period.

*General observations on hybrid clones subcultured as cell lines*

Selected colonies were subcultured from day 21 onwards (Methods), for further characterisation. They were selected initially for differences in colony size, pigmentation and morphology, and from cultures originally grown with and without TPA. Cell morphology of most hybrids proved however to vary with cell density and perhaps passage number, and could not therefore be used reliably to classify hybrid cell lines. Pigmentation also varied with cell density in melan-b/B16-F10$^{RR}$ hybrids as just described.

It should be stressed that the 19 hybrid lines studied cannot necessarily be taken as a completely representative sample of the thousands of original hybrids, especially given that many of the latter failed to grow progressively at all (Figure 2).

*Proliferation rates of hybrid lines and effects of TPA*

Lines from hybrid colonies selected initially in the presence or the absence of TPA were each passaged into media both with and without TPA. Estimates of crude doubling times over 4 days showed that all hybrids grew more slowly than the melanoma cells under both conditions, while most melanoma/melanocyte hybrids grew faster than the parental melanocyte lines (Table I). All tested hybrid clones showed a stimulation of growth by TPA, except one; this one (F7.11B) was a melan-b/B16-F10 hybrid initially obtained without TPA (Table I).

The melanocyte/melanocyte hybrids had doubling times comparable with those of the parental lines in the presence of TPA. When TPA was excluded from the culture medium their rate of proliferation slowed markedly over 4 days (Table I), and they adopted a more epithelioid morphology, also characteristic of melanocyte lines (Bennett *et al.*, 1987, 1989).

The effects of TPA on the B16-F10$^{RR}$ parental line were also examined, as this had not been done previously. Proliferation of these cells over the first 4 days in the presence of TPA was slightly inhibited, their doubling time being 0.92 days compared with 0.85 days in its absence. However other

**Figure 3** Melan-b/B16-F10$^{RR}$ hybrids. The cells were cultured as in Materials and methods and photographed through an Olympus inverted microscope. **a**, Pigmented clone selected in the absence of TPA. Bright field illumination; scale bar 200 μm. **b**, F7.19A hybrid, unpigmented when initially selected in the presence of TPA, showing reversion to pigmentation at low cell density after culture for 5 days in the absence of TPA. Phase contrast illumination; scale bar 200 μm.

**Table I** Effect of TPA on adherent growth of hybrid and parental cell lines

| *Cell line* | *Doubling time of adherent cultures (days)*[a] *+TPA* | *−TPA* |
|---|---|---|
| Parental cell lines: | | |
| B16-F10$^{RR}$ | 0.92 | 0.85 |
| melan-c | 2.98 | 5.45 |
| melan-b | 2.46 | [b] |
| melan-c/B16-F10$^{RR}$ hybrids[c]: | | |
| F5.3A | 1.34 | 2.19 |
| F5.6A | 1.65 | 2.74 |
| F5.7A | 1.39 | 3.88 |
| F5.22A | 1.31 | 1.6 |
| F5.1B | 2.57 | 3.52 |
| F5.4B | 1.91 | 3.55 |
| F5.5B | 1.26 | 1.55 |
| F5.8B | 2.06 | 2.78 |
| melan-b/B16-F10$^{RR}$ hybrids: | | |
| F7.14A | 1.55 | 1.93 |
| F7.19A | 2.38 | 3.98 |
| F7.21A | 1.8 | 2.32 |
| F7.4B | 1.52 | 1.84 |
| F7.5B | 1.28 | 1.66 |
| F7.8B | 1.54 | 1.97 |
| F7.11B | 1.47 | 1.18 |
| melan-c/melan-b$^{HT}$ hybrids: | | |
| F9.1A | 3.38 | 5.66 |
| F9.2A | 2.49 | 6.29 |
| F9.3A | 3.01 | ∞[d] |
| F9.5A | 2.37 | 7 |

[a]Crude doubling times over 4 days on plastic were measured as in Materials and methods. [b]Not done. Melan-b cells grew poorly in the absence of TPA (Bennett *et al.*, 1989). [c]Hybrid lines designated A and B were selected originally in the presence and absence of TPA, respectively. [d]No significant change in cell number.

**Table II** Effect of TPA on non-adherent clonogenic growth of hybrid and parental cell lines

| *Cell line* | *Cloning efficiency in suspension (%)*[a,b] *+TPA* | | *−TPA* | |
|---|---|---|---|---|
| Parental cell lines: | | | | |
| B16-F10$^{RR}$ | 5.7 | (0.12) | 52 | (0.3) |
| melan-c | 0 | | 0 | |
| melan-b | 0 | | 0 | |
| melan-c/B16-F10$^{RR}$ hybrids: | | | | |
| F5.3A | 48 | (0.06) | 0.75 | (0.05) |
| F5.6A | 1 | (0.05) | 0 | |
| F5.7A | 2.5 | (0.05) | 0 | |
| F5.22A | 37 | (0.09) | 0 | |
| F5.1B | 17 | (0.1) | 14 | (0.06) |
| F5.4B | 0.83 | (0.05) | 0.33 | (0.05) |
| F5.5B | 3.83 | (0.05) | 0 | |
| F5.8B | 22 | (0.11) | 10 | (0.08) |
| melan-b/B16-F10$^{RR}$ hybrids: | | | | |
| F7.14A | 1.5 | (0.05) | 0 | |
| F7.19A | 0 | | 0 | |
| F7.21A | 0 | | 0 | |
| F7.4B | 0.83 | (0.11) | 0.33 | (0.1) |
| F7.5B | 0.83 | (0.05) | 0.33 | (0.05) |
| F7.8B | 0 | | 0 | |
| F7.11B | 0 | | 0 | |
| melan-c/melan-b$^{HT}$ hybrids: | | | | |
| F9.1A | 0 | | 0 | |
| F9.2A | 0 | | 0 | |
| F9.3A | 0 | | 0 | |
| F9.5A | 0 | | 0 | |

[a]Cloning efficiencies over 21 days in soft agarose were measured as in Materials and methods. [b]Figures in brackets represent the mean colony diameter (mm). Differences in colony sizes between hybrids grown with and without TPA were not significant by the chi-squared test ($P<0.1$).

work with B16-F10 cells (non-drug-resistant) showed that after two subcultures (7 days) in the presence of TPA, their growth rate with TPA over the next 4 days was markedly lower, typical doubling times being 1.0–1.1 days, compared with 0.65–0.7 days without TPA for this line (increases of around 15-fold instead of 60-fold over 4 days).

### *Clonogenicity of hybrid lines in semisolid agarose, and effects of TPA*

The percentages of cells forming colonies in suspension in two-phase agarose, for parental and hybrid lines, are shown in Table II. The two melanocyte lines and all hybrids between them formed no colonies in suspension, with or without TPA, while the clonogenicity of the parental melanoma cells was high (52%) in the absence of TPA, or lower (6%) in its presence. The melanocyte/melanoma hybrid lines varied in their capacity to grow in suspension. Among melan-b/B16 hybrids, most (4/7) formed no colonies, two had cloning efficiencies below 1% with or without TPA and the remaining line had clonogenicities of 0 in the absence of TPA and 1.5% in its presence. Thus the ability of melan-b/B16 hybrids to grow in suspension was zero or markedly lower than that of the melanoma cells; where not zero it may have been stimulated slightly by TPA. Of eight melan-c/B16 hybrid lines tested, most showed different behaviour with and without TPA. In the absence of TPA, four lines were unable to form suspension colonies, two showed less than 1% clonogenicity and the other two formed significant numbers of colonies (14%, 10%). However when TPA was present, all hybrids formed some colonies. Four had cloning efficiencies of 3% or less, while those of the other four were 17% or more. Thus for this set of hybrids, the clonogenicity in agarose tended to be intermediate between those of the parental lines, although generally near zero in the absence of TPA. Where hybrids formed colonies both with and without TPA, most had marginally larger mean diameters in the presence of TPA but this was not significant by the chi-squared test ($P > 0.1$).

*Tumorigenicity of hybrid lines*

Hybrid lines were selected for tests of tumorigenicity in animals on the basis of either high or low cloning efficiencies in suspension. This was to test whether the two properties were correlated, as reported for other cell types by some authors (e.g. Freedman & Shin, 1974). Cells at passage 5 were implanted into nude and C57BL/6 mice, the latter syngeneic with B16 cells. For comparison, the melan-b and melan-c lines formed no tumours in 6 months after implantation of $2 \times 10^6$ cells in each of ten mice per line (Bennett *et al.*, 1989), while B16-F10$^{RR}$ cells were tumorigenic in 100% of syngeneic mice within 4 weeks from an inoculum of $5 \times 10^4$ cells (Table III) or even within 2 weeks, from an inoculum of $10^5$ cells (I.R. Hart, personal communication).

A total of 4 melan-c/B16-F10$^{RR}$ and 4 melan-b/B16-F10$^{RR}$ hybrids were examined for tumorigenicity (Table III). Four hybrids, namely two melan-c/B16-F10$^{RR}$ (F5.3A and F5.6A) and two melan-b/B16$^{RR}$ hybrids (F7.21A and F7.5B) showed a marked reduction in tumorigenicity. Latent periods were generally long and some mice developed no tumour in the test period. Although the remaining hybrids produced tumours in both *nude* and C57BL/6 mice in 4–6 weeks, their tumorigenicity was still distinctly lower than that of the melanoma line, since not all mice had tumours at 4 weeks from a relatively large inoculum. From the hybrid lines examined, there appeared to be no difference in the ability of melan-c and melan-b cells to suppress the tumorigenicity of the melanoma. There was no evidence for a correlation between tumorigenicity of the hybrids and their growth in semisolid medium (note L and H designations, Table III), although sample sizes were small.

*Karyology*

Modal chromosome numbers of 40 (diploid) for melan-c and 39 for melan-b cells were established previously from 50 spreads per line, from cultures at passage levels near those used in the present experiments (Bennett *et al.*, 1989). The present banding studies failed to reveal marker chromosomes in either melanocyte line. B16-F10$^{RR}$ cells had a narrow range of chromosome numbers (75–77, mode = 76) (near tetraploid). Several marker chromosomes were found, including a Robertsonian translocation between chromosomes 12, a metacentric chromosome 10 with additional unidentified non-centromeric DNA and other small unidentified metacentric chromosomes.

Spreads were prepared from 10 melanocyte/melanoma hybrid lines at passage 3. These did not generally have distinct modal chromosome numbers. Melan-c/B16 hybrids (F5 designations) generally had wider ranges than melan-b/B16 hybrids (F7 designations), but mean numbers of chromosomes were similar. Actual means and ranges were:- F5.3A, 94.2 (64–104); F5.6A, 95.2 (57–116); F5.7A, 103.9 (92–136); F5.22A [passage 5], 84.2 (63–98). F7.19A, 100.3 (98–104); F7.21A, 95.2 (85–104); F7.4B, 95.3 (76–106); F7.5B, 95.3 (64–127); F7.8B, 93.3 (76–98); F7.11B, 101 (96–106).

These numbers and the presence and numbers of B16-F10$^{RR}$ marker chromosomes in each hybrid suggested that all the hybrids examined had originally received a single nucleus from each parental line. Chromosome loss was thus quite extensive in all hybrids, even at the earliest possible stage for study after fusion (passage 3).

### Discussion

*Pigmentation*

The pigmentation of the melanocyte/melanoma hybrid clones was most interesting. It appeared that all melan-c/B16 hybrid cells were black-pigmented, so that the differentiated state of the normal melanocytes was unequivocally dominant here. The same can be said of melan-b/B16 hybrid cells cultured without TPA. However the latter hybrids in the presence of TPA behaved somewhat like B16 melanoma cells grown without TPA: pigmentation was absent in sparse cultures but appeared in locally dense areas. We have no explanation for this difference between the two sets of hybrids. It presumably reflects a difference between the melan-b and melan-c lines, which probably arose during their establishment rather than

**Table III** Tumorigenicity of hybrid cell lines

| Cell line | Tumour incidence[a] C57BL/6 mice 4 wk | 6 wk | 12 wk | BALB/c nu/nu mice 4 wk | 6 wk | 11 wk | Summary[b] |
|---|---|---|---|---|---|---|---|
| B16-F10$^{RR}$ | 5/5 | | | ND | | | + + + |
| melan-c/B16-F10$^{RR}$ hybrids: | | | | | | | |
| F5.3A (H)[c] | 0/5 | 1/5 | 4/5 | 0/5 | 0/5 | 3/4 | + |
| F5.6A (L) | 0/2 | 0/2 | 0/2 | 0/3 | 0/1 | 1/1 | −/+ |
| F5.7A (L) | 2/5 | 3/5 | 5/5 | 1/5 | 4/4 | 4/4 | + + |
| F5.22A (H) | 1/5 | 5/5 | 5/5 | 1/4 | 4/4 | 4/4 | + + |
| melan-b/B16-F10$^{RR}$ hybrids: | | | | | | | |
| F7.19A (L) | 4/4 | 4/4 | 4/4 | 2/3 | 2/3 | 2/3 | + + |
| F7.21A (L) | 0/4 | 1/4 | 1/4 | 0/4 | 0/4 | 3/4 | + |
| F7.4B (L) | 4/4 | 4/4 | 4/4 | 3/4 | 3/3 | 3/3 | + + |
| F7.5B (L) | 0/4 | 1/4 | 2/4 | 0/4 | 1/4 | 4/4 | + |

[a]Number of mice with tumours out of total surviving mice, assessed as described in Materials and methods using $10^6$ hybrid cells per mouse, or $5 \times 10^4$ B16-F10$^{RR}$ melanoma cells. ND: not done; hybrid lines were tested in both C57BL/6 and *nu/nu* mice in case they were rejected by the C57BL/6 mice, whereas B16-F10$^{RR}$ cells were tested only in C57BL/6 mice with which they are syngeneic. [b]Summary of tumorigenicity: (−): no tumours in 12 weeks; (+): low tumorigenicity – under 30% mice with tumours in 6 weeks; (+ +): tumorigenic but < 100% in 4 weeks; (+ + +): highly tumorigenic – 100% in 4 weeks (or in 2 weeks; see text), from a much smaller inoculum. [c](L) and (H) designate low and high clonogenicities in suspension, respectively (see Table II).

from the mouse strains of origin. The melan-c/melanoma hybrids did appear less pigmented when growing with TPA than without, but did not become unpigmented. Possibly melan-b cells are in some sense less maturely differentiated than melan-c cells, producing a corresponding difference in their hybrids with B16-F10 cells. Human melanoma cells have been classified into three sets with different levels of differentiation (Houghton *et al.*, 1987); however both melan-b and melan-c cells would fall into the most-differentiated class in this system, since they synthesise melanosomes and melanosomal enzymes.

These results are comparable with other reports that when non-tumorigenic cells with a differentiated function, such as keratinocytes, are fused to malignant cells of another lineage, the hybrids before extensive chromosome loss tend to adopt the program of differentiation of the non-tumorigenic fusion partner, this behaviour usually being associated with a lack of tumorigenicity (Wiener *et al.*, 1972; Stanbridge & Ceredig, 1981; Cowell & Franks, 1984; Harris, 1990), although not always (Cowell & Franks, 1984). An early report of two hybrid lines, formed by spontaneous fusion *in vivo* between subtetraploid mouse melanoma cells and unknown diploid host cell types(s), mentioned that both lines, while tumorigenic, showed increased pigment synthesis in culture compared to the melanoma cells (Halaban *et al.*, 1980).

An incidental outcome of the pigmentation of our hybrid cells was that we could identify them at a very early stage, as groups of one or a few black cells. This permitted the additional observation that many hybrids, in fact the majority of melan-c/B16 hybrids (Figure 2), failed to divide in 14 days, or apparently stopped growing after very few divisions. This may have been due to cell-cycle incompatibilities, loss of vital chromosomes, drug effects, gene dosage effects or perhaps to complementation between different mutations producing immortality, resulting in senescence of hybrid cells, as reported for hybrids between human cell lines (Pereira-Smith & Smith, 1988). In the last case, chromosome loss would be necessary for any progressive hybrid cell proliferation. This observation reinforces the view that the properties of the hybrid lines analysed may not have been fully representative of the original hybrid population; the lines may well have been less 'normal' than average. In most other such studies, of course, the only hybrid cells detected have been those that grew progressively.

*Proliferation and effects of TPA*

In the proliferation rates of the hybrid cells in standard (adherent) cultures, the behaviour of neither the malignant nor the non-malignant cells was completely dominant. All hybrids that were examined as cell lines were able to grow in the absence of TPA, like melanoma cells and unlike melanocytes, but their growth rates without TPA were generally markedly lower than those of the melanoma cells. Proliferation rates of hybrid lines in the presence of TPA were also generally intermediate between those of the melanocytes and the melanoma cells. One interpretation is that proliferation rates are determined by more than one gene, and among these genes, dominance of one or more melanocyte genes is offset by dominance of one or more melanoma genes. On the other hand, fewer growing colonies were originally obtained in the absence than in the presence of TPA after fusion of melan-c and B16 cells; this suggests that initially, at least, some hybrid cells were present that could grow in the presence but not the absence of TPA. It should be noted that faster-growing subpopulations arising in a clone through loss of melanocyte-derived chromosomes could soon come to predominate numerically.

Perhaps more striking was the direction of the proliferative response of each hybrid to TPA. All melan-c/B16 hybrids and all but one melan-b/B16 hybrid tested grew faster with TPA than without, like melanocytes and unlike melanoma cells. This is thus another property of the non-tumorigenic cells that behaves dominantly. The exceptional clone (F7.11B) had been obtained in the absence of TPA, so selective pressure could have led to loss of relevant chromosome(s) derived from the melanocyte parent line.

*Growth in suspension*

The high clonogenicity in agarose of the B16-F10$^{RR}$ melanoma cells was suppressed completely in some hybrids with melan-b, and in some hybrids with melan-c so long as TPA was absent. Other hybrids grew in suspension to various extents, though none grew in the absence of TPA so well as the melanoma cells. The growth rates of different hybrid lines on a substrate with and without TPA did not correlate well with their growth in agarose, so the stimulation of hybrid cell growth in suspension by TPA cannot be attributed in a straightforward way to its ability to stimulate cell proliferation. In general the variability in growth in agarose within and between the lines may be attributable to variable loss of relevant chromosome(s). However suppression of melanoma growth in agarose appeared more marked in hybrids with melan-b than with melan-c cells, even though rates of chromosome loss by passage 3 appeared to have been similar in both sets in those hybrids karyotyped. This suggests instead another pre-existing difference between the two melanocyte lines.

*Tumorigenicity*

There is a consensus from previous studies that hybrids between non-tumorigenic and tumorigenic cells (including melanoma cells) tend to be non-tumorigenic before chromosome loss (e.g. Stanbridge & Ceredig, 1981; Harris, 1990). Our results are consistent with this idea; the observation of some mice free of tumours at the end of the assay period, and the often long latent periods, indicate that in general the tumours arose from a small minority of the implanted cells. Tumorigenicity would be below 1 cell in $10^6$ for lines not tumorigenic in all mice. This represents almost complete suppression as compared to B16-F10$^{RR}$ cells, of which at least 1 in 50,000 is tumorigenic from Table III, and at least 1 in 500 in the tests of Hart (1984) (under different conditions). Considerable chromosome loss had occurred by the passage (number 5) at which enough cells were available for implantation in mice, so that the tumours observed can be explained by the loss of chromosome(s) necessary for suppression. The observation that no tested hybrid was as tumorigenic as the melanoma cells suggest that more than one gene from the melanocytes was capable of reduction of tumour growth.

Incidentally a suppression of tumorigenicity is not caused by hybridisation itself in these cells. Hart (1984) fused B16-F10$^{RR}$ cells to various other cell lines and observed a reduction of malignancy only when the other line was non-tumorigenic. Similar results were obtained by others; for example 16/16 hybrid lines from combinations of various other tumorigenic murine lines were tumorigenic (Wiener *et al.*, 1973), and human tumour lines too remained tumorigenic when cross-hybridised (Stanbridge *et al.*, 1982).

None of the other cellular properties analysed (pigmentation at low pH, response to TPA and growth in agarose) was a predictor of the tumorigenicity of these hybrid clones, but this finding is compromised by the cellular heterogeneity resulting from variable chromosome loss. The properties measured *in vitro* are those of the majority cell population, but the tumorigenicity depends on the most tumorigenic cells present, often a small subpopulation as just mentioned, of which the properties *in vitro* would not be known – e.g. growth in suspension of 1 in $10^6$ cells would not be detected. Moreover, tumorigenicity tests were done two passages later than the other tests, allowing further chromosome loss. Each of the said properties *is* correlated with cellular malignancy among pigment cell lines in general (see Introduction).

*Implications for melanoma genetics*

In short, of the properties studied in these hybrid cells, most seemed closer to those of the non-tumorigenic melanocytes than those of the melanoma cells, and chromosome loss was sufficient to account for most deviations from this rule, even though the melanocytes used here were immortal rather than fully normal. It is possible that a still higher degree of 'normalisation' would have been seen with newly explanted, diploid murine melanocytes, which, however, are difficult to work with because of their rapid senescence and overgrowth by immortal cells.

In a detailed cytogenetic study of hybrids between a murine melanoma and diploid fibroblasts or lymphocytes, it was concluded that tumour suppression was seen only when a hybrid retained one or both copies of the normal mouse chromosome 4 (Jonasson *et al.*, 1977). Copies of normal chromosome 4 were also implicated in suppression of malignancy of other types of tumour, and evidence was presented that the ratio between numbers of normal and tumour-derived chromosomes 4 was important in whether suppression was observed (Evans *et al.*, 1982). The capacity for suppression was localised to a specific region of this chromosome (Jonasson *et al.*, 1977), restated to be bands A4-C3 (Harris, 1990). This contains a region of homology to human chromosome 1, arm 1p, chromosome 1 being associated with suppression of malignancy of a hamster cell line (Stoler & Bouck, 1985; Harris, 1990). There is also a small region syntenic with human chromosome 6q (Ceci *et al.*, 1989). Human chromosome 6 has been directly shown, by microcell-mediated chromosome transfer, to suppress malignancy of two human melanoma lines in *nude* mice (Trent *et al.*, 1990). Human chromosome arms 1p and 6q are those most commonly subject to deletions and translocations in melanomas (Trent *et al.*, 1989). One group has mapped a familial susceptibility to cutaneous melanoma to human chromosome 1p36 (Bale *et al.*, 1989), although other groups have reported conflicting data. All these findings suggest the loss of specific normal genes in melanoma.

Here the genetic analysis was extended to cellular properties observed *in vitro*. It is concluded that, here too, properties of the normal pigment cells tend to be dominant over those of melanoma cells, the most reliable being pigmentation and a positive growth response to TPA. These findings are of considerable practical value, providing two relatively rapid assays for the normal cellular gene or genes responsible. These assays will permit attempts at chromosome-mediated or DNA-mediated transfer of these biological properties from normal cells to melanoma cells. Any sequence capable of conferring one or both properties would be most interesting, not only as a candidate melanoma suppressor gene.

We are greatly indebted to Dr Ian Hart of the Imperial Cancer Research Fund, London for supplying the B16-F10 and B16-F10$^{RR}$ cell lines, and for carrying out the tumorigenicity tests; also to Drs Ian Jackson and Robin Allshire (MRC Human Genetics Unit, Edinburgh) for the pUC-Y3 plasmid. This work was supported by the Cancer Research Campaign.

### References

ALBINO, A., HOUGHTON, A.N., EISINGER, M. & 4 others (1986). Class II histocompatibility antigen expression in human melanocytes transformed by Harvey murine sarcoma virus (Ha-MSV) and Kirsten MSV retroviruses. *J. Exp. Med.*, **164**, 1710.

BALE, S.J., DRACOPOLI, N.C., TUCKER, M.A. & 7 others (1989). Mapping the gene for hereditary cutaneous malignant melanoma-dysplastic nevus to chromosome 1p. *New England J. Med.*, **320**, 1367.

BENNETT, D.C., COOPER, P.J., DEXTER, T.J., DEVLIN, L.M., HEASMAN, J. & NESTER, B. (1989). Cloned mouse melanocyte lines carrying the germline mutations albino and brown: complementation in culture. *Development*, **105**, 379.

BENNETT, D.C., COOPER, P.J. & HART, I.R. (1987). A line of non-tumorigenic mouse melanocytes, syngeneic with the B16 melanoma and requiring a tumour promoter for growth. *Int. J. Cancer*, **39**, 414.

BENNETT, D.C., DEXTER, T.J., ORMEROD, E.J. & HART, I.R. (1986). Increased experimental metastatic capacity of a murine melanoma following induction of differentiation. *Cancer Res.*, **46**, 3239.

BLOCHLINGER, K. & DIGGELMANN, H. (1984). Hygromycin B phosphotransferase as a selectable marker for DNA transfer experiments with higher eucaryotic cells. *Mol. & Cell. Biol.*, **4**, 2929.

CECI, J.D., SIRACUSA, L.D., JENKINS, N.A. & COPELAND, N.G. (1989). A molecular genetic linkage map of mouse chromosome 4 including the localization of several proto-oncogenes. *Genomics*, **5**, 699.

COOK, P.J, DOLL, R. & FELLINGHAM, S.A. (1969). A mathematical model for the age distribution of cancer in man. *Int. J. Cancer*, **4**, 93.

COOPER, J.A. (1990). Oncogenes and anti-oncogenes. *Curr. Opinion Cell Biol.*, **2**, 285.

COWELL, J.K. & FRANKS, L.M. (1984). The ability of normal cells to reduce the malignant potential of transformed mouse bladder epithelial cells depends on their somatic origin. *Int. J. Cancer*, **33**, 657.

DOTTO, G.P., MOELLMANN, G., GHOSH, S., EDWARDS, M. & HALABAN, R. (1989). Transformation of murine melanocytes by bFGF cDNA and selective suppression of the transformed phenotype in a reconstituted cutaneous environment. *J. Cell. Biol.*, **109**, 3115.

EISINGER, M. & MARKO, O. (1982). Selective proliferation of normal human melanocytes in vitro in the presence of phorbol ester and cholera toxin. *Proc. Natl. Acad. Sci. USA*, **79**, 2018.

EVANS, E.P., BURTENSHAW, M.D., BROWN, B.B., HENNION, R. & HARRIS, H. (1982). The analysis of malignancy by cell fusion. IX. Re-examination and clarification of the cytogenetic problem. *J. Cell Sci.*, **56**, 113.

FIDLER, I.J. (1975). Biological behavior of malignant melanoma cells correlated to their survival *in vivo*. *Cancer Res.*, **35**, 218.

FOUGERE, C., RUIZ, F. & EPHRUSSI, B. (1972). Gene dosage dependence of pigment synthesis in melanoma × fibroblast hybrids. *Proc. Natl Acad. Sci. USA*, **69**, 330.

FREEDMAN, V.H. & SHIN, S. (1974). Cellular tumorigenicity in *nude* mice: correlation with cell growth in semi-solid medium. *Cell*, **3**, 355.

HALABAN, R., NORDLUND, J., FRANCKE, U., MOELLMANN, G. & EISENSTADT, J.M. (1980). Supermelanotic hybrids derived from mouse melanomas and normal mouse cells. *Somat. Cell Genet.*, **6**, 29.

HARRIS, H. (1990). The role of differentiation in the suppression of malignancy. *J. Cell Sci.*, **97**, 5.

HARRIS, H., MILLER, O.J., KLEIN, G., WORST, P. & TACHIBANA, T. (1969). Suppression of malignancy by cell fusion. *Nature*, **223**, 363.

HART, I.R. (1984). Tumor cell hybridization and neoplastic progression. In *Cancer Invasion and Metastasis: Biologic and Therapeutic Aspects*, Nicolson, G.L. & Milas, L. (eds) p. 133. Raven Press, New York.

HERLYN, M., KATH, R., WILLIAMS, N., VALYI-NAGY, I. & RODECK, U. (1990). Growth-regulatory factors for normal, premalignant, and malignant human cells in vitro. *Adv. Cancer Res.*, **54**, 213.

HOUGHTON, A.N., REAL, F.X., DAVIS, L.J., CORDON-CARDO, C. & OLD, L.J. (1987). Phenotypic heterogeneity of melanoma: relation to the differentiation program of melanoma cells. *J. Exp. Med.*, **164**, 812.

HUANG, H.-J.S., YEE, J.-K., SHEW, J.-Y. & 5 others (1988). Suppression of the neoplastic phenotype by replacement of the RB gene in human cancer cells. *Science*, **242**, 1563.

JONASSON, J., POVEY, S. & HARRIS, H. (1977). The analysis of malignancy by cell fusion. VII. Cytogenetic analysis of hybrids between malignant and diploid cells and of tumours derived from them. *J. Cell Sci.*, **24**, 217.

LASKIN, J.D., MUFSON, R.A., WEINSTEIN, I.B. & ENGELHARDT, D.L. (1980). Identification of a distinct phase during melanogenesis that is sensitive to extracellular pH and ionic strength. *J. Cell. Physiol.*, **103**, 467.

LITTLEFIELD, J.W. (1964). Selection of hybrids from matings of fibroblasts in vitro and their presumed recombinants. *Science*, **145**, 709.

MUSCHEL, R.J., NAKAHARA, K., CHU, E., POZATTI, R. & LIOTTA, L.A. (1986). Karyotypic analysis of diploid or near diploid metastatic Harvey ras transformed rat embryo fibroblasts. *Cancer Res.*, **46**, 4104.

NIGRO, J.M., BAKER, S.J., PREISINGER, A.C. & 13 others (1989). Mutations in the p53 gene occur in diverse human tumour types. *Nature*, **342**, 705.

PEREIRA-SMITH, O.M. & SMITH, J.R. (1988). Genetic analysis of indefinite division in human cells: identification of four complementation groups. *Proc. Natl Acad. Sci. USA*, **85**, 6042.

SATO, C., ITO, S. & TAKEUCHI, T. (1985). Establishment of a mouse melanocyte clone which synthesizes both eumelanin and pheomelanin. *Cell. Struct. Funct.*, **10**, 421.

STANBRIDGE, E.J. & CEREDIG, R. (1981). Growth-regulatory control of human cell hybrids in nude mice. *Cancer Res.*, **41**, 573.

STANBRIDGE, E.J., DER, C.J., DOERSEN, C.-J. & 4 others (1982). Human cell hybrids: analysis of transformation and tumorigenicity. *Science*, **215**, 252.

STOLER, A. & BOUCK, N. (1985). Identification of a single chromosome in the normal human genome essential for suppression of hamster cell transformation. *Proc. Natl Acad. Sci. USA*, **82**, 570.

TAMURA, A., HALABAN, R., MOELLMANN, G., COWAN, J.M., LERNER, M.R. & LERNER, A.B. (1987). Normal murine melanocytes in culture. *In Vitro Cell. Dev. Biol.*, **23**, 519.

TRENT, J.M., LEONG, S.P.L. & MEYSKENS, F. (1989). Chromosome alterations in malignant melanoma. *Carcinog. Compr. Surv.*, **11**, 165.

TRENT, J.M., STANBRIDGE, E.J., MCBRIDE, H.L. & 5 others (1990). Tumorigenicity in human melanoma cell lines controlled by introduction of human chromosome 6. *Science*, **247**, 568.

WIENER, F., COCHRAN, A., KLEIN, G. & HARRIS, H. (1972). Genetic determinants of morphological differentiation in hybrid tumors. *J. Natl Cancer Inst.*, **48**, 465.

WIENER, F., KLEIN, G. & HARRIS, H. (1973). The analysis of malignancy by cell fusion. IV. Hybrids between tumour cells and a malignant L-cell derivative. *J. Cell Sci.*, **12**, 253.